# Experimental Validation and Evaluation of the Bending Properties of Additively Manufactured Metallic Cellular Scaffold Structures for Bone Tissue Engineering

**DOI:** 10.3390/ma15103447

**Published:** 2022-05-11

**Authors:** Mohammad O. Al-Barqawi, Benjamin Church, Mythili Thevamaran, Dan J. Thoma, Adeeb Rahman

**Affiliations:** 1 Department of Civil and Environmental Engineering, University of Wisconsin, Milwaukee, WI 53211, USA; adeeb@uwm.edu; 2 Department of Material Science and Engineering, University of Wisconsin, Milwaukee, WI 53211, USA; church@uwm.edu; 3 Department of Material Science and Engineering, University of Wisconsin, Madison, WI 53706, USA; mthevamaran@wisc.edu (M.T.); dthoma@wisc.edu (D.J.T.)

**Keywords:** cellular structures, additive manufacturing, SS 316L scaffolds, bending properties

## Abstract

The availability of additive manufacturing enables the fabrication of cellular bone tissue engineering scaffolds with a wide range of structural and architectural possibilities. The purpose of bone tissue engineering scaffolds is to repair critical size bone defects due to extreme traumas, tumors, or infections. This research study presented the experimental validation and evaluation of the bending properties of optimized bone scaffolds with an elastic modulus that is equivalent to the young’s modulus of the cortical bone. The specimens were manufactured using laser powder bed fusion technology. The morphological properties of the manufactured specimens were evaluated using both dry weighing and Archimedes techniques, and minor variations in the relative densities were observed in comparison with the computer-aided design files. The bending modulus of the cubic and diagonal scaffolds were experimentally investigated using a three-point bending test, and the results were found to agree with the numerical findings. A higher bending modulus was observed in the diagonal scaffold design. The diagonal scaffold was substantially tougher, with considerably higher energy absorption before fracture. The shear modulus of the diagonal scaffold was observed to be significantly higher than the cubic scaffold. Due to bending, the pores at the top side of the diagonal scaffold were heavily compressed compared to the cubic scaffold due to the extensive plastic deformation occurring in diagonal scaffolds and the rapid fracture of struts in the tension side of the cubic scaffold. The failure in struts in tension showed signs of ductility as necking was observed in fractured struts. Moreover, the fractured surface was observed to be rough and dull as opposed to being smooth and bright like in brittle fractures. Dimple fracture was observed using scanning electron microscopy as a result of microvoids emerging in places of high localized plastic deformation. Finally, a comparison of the mechanical properties of the studied BTE scaffolds with the cortical bone properties under longitudinal and transverse loading was investigated. In conclusion, we showed the capabilities of finite element analysis and additive manufacturing in designing and manufacturing promising scaffold designs that can replace bone segments in the human body.

## 1. Introduction

The availability of additive manufacturing (AM) enables the fabrication of cellular bone tissue engineering (BTE) scaffolds with a wide range of structural and architectural possibilities [1,2,3,4]. Moreover, AM provides the ability to design scaffolds with a specific geometry and bone mimicking mechanical properties [5] to minimize the stress shield phenomenon, which is the main cause of bone weakening and reduction in bone density due to stiffness mismatch between the scaffold and surrounding bone tissues.

The purpose of BTE scaffolds is to repair critical size bone defects due to extreme traumas, tumors, or infections [6,7]. Clinical applications include the repair of open tibial shaft fractures and large femoral segment bone defects [8]. Consequently, bone scaffolds should satisfy specific mechanical and biological requirements to facilitate bone healing. Scaffolds should be osteoconductive, osteoinductive, mechanically compatible, and made of a biocompatible material.

Scaffolds are made from ceramic, polymeric, or metallic materials. Metallic materials are commonly used as biomaterials such as magnesium (Mg) and titanium (Ti) alloys due to their excellent biocompatibility, corrosion resistance, non-toxicity, and excellent mechanical properties such as compressive strength, fatigue resistance, and fracture toughness. However, scaffold materials such as magnesium undergo rapid degradation. This early degradation rate results in a weakening of the mechanical strength of the scaffold. Coatings improve the corrosion rate of metallic scaffolds and enhance bioactivity and osteointegration [9]. The recent advances in coating materials of Mg alloy, including metals, inorganic non-metals, polymers, and composite coatings, as well as their effects on corrosion resistance and biocompatibility, were reviewed by [10].

The scaffold structure is an open-cell foam that can be formed by replicating polyhedral unit cells. Eleven types of unit cells were examined and presented [11,12], including triangular prism, square prism, hexagonal prism, octagonal prism, cuboctetradron, truncated octahedron, truncated cube, Rhombicuboctehedron, truncated cuboctetradron, rhombic dodecahedron, and square pyramid. Furthermore, other unit cells such as cubic lattice [13], diamond lattice, truncated cube [14], the truncated octahedron [15], the rhombic dodecahedron [16], and rhombicuboctahedron [16], were explored. At this point, there is no general rule for selecting the best unit cell type to manufacture the BTE scaffold.

In [17] study, numerical optimization was conducted to design bone tissue scaffolds with a structural modulus similar to the cortical bone modulus to minimize the stress shielding phenomenon. The pore size was fixed at 800 μm, which is sufficient to allow bone ingrowth inside the scaffold, according to studies presented by [18,19,20]. Cubic and diagonal unit cells were considered. The scaffolds were additively manufactured using laser powder bed fusion (LPBF) technology and experimentally tested in compression. The experimental results were observed to agree with the numerical findings. More details on the optimization procedure are presented in [17] study.

This study aims to experimentally validate and evaluate the bending and shear properties of the designed scaffolds presented in [16] study. The bending and shear moduli were numerically calculated using finite element analysis (FEA). A three-point bending test was conducted on additively manufactured beam specimens to validate the numerical results and evaluate the failure mechanism for the cubic and diagonal scaffold designs. Similar work was conducted by [21] on Ti-6Al-4V scaffolds, which was limited to numerical analysis without experimental validation. [22] studied the compressive, bending, and torsional properties of cubic, diamond, and body-centered cubic (BCC) designs. BCC. BCC was observed to exhibit isotropic mechanical properties and mimic bone properties of the porous stem.

## 2. Material and Methods

### 2.1. BTE Scaffold Material

The material used for this study is stainless steel (SS) 316L since it is one of the most common metallic BTE scaffold materials and is widely used in AM. SS 316L (EOS, Munich, Germany) coupon was additively manufactured using the SLM process and tested by [23]. The obtained mechanical properties of the 3D-printed SS material are shown in Table 1. The same properties were used in the FEA study to numerically evaluate the bending and shear moduli of the cubic and diagonal scaffolds.

### 2.2. Numerical Computation of the Bending and Shear Moduli of the BTE Scaffolds

**Figure 1:**
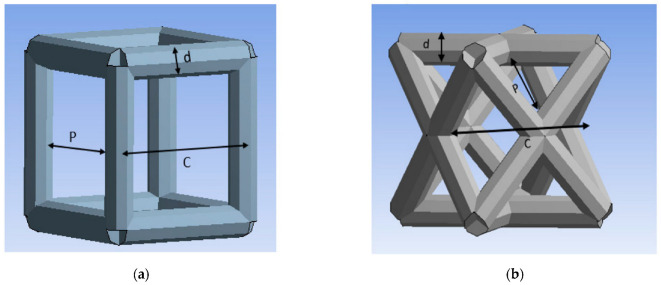
Unit cells: (**a**) cubic, (**b**) diagonal.

ANSYS software (ANSYS 2021 R2, John A. Swanson, Canonsburg, PA, USA) has been used as the FEA tool. The unit cell types investigated in this study are the cubic and diagonal cell shapes. Each unit cell has three geometrical parameters: cell size (c), strut size (d), and pore size (p), as shown in Figure 1.

The final geometrical parameters generated from the direct optimization conducted by [17] targeting the structural modulus of the cortical bone are presented in Table 2.

Beams were created using the results from the optimization and modeled using FEA in bending and torsion. The beams are fixed from one end, and deformation is applied at the other end. Vertical deflection is applied at the free end to simulate bending, and free end rotation is applied to simulate torsion. The bending and shear moduli are calculated using the following relationships:

(1) $$E_{b}^{*}=\frac{F_{R}*L_{O}}{3*I\;*\delta }$$

(2) $$G^{*}=\frac{T_{R}\;*L_{O}}{3*J\;*\theta }$$

where *F_R_* is the reaction at the fixed end support, *L_o_* is the beam length, *I* is the moment of inertia, *δ* is the deflection at the free end, *T_R_* is the torque reaction at the end support, *J* is the polar moment of inertia, and *θ* is the free end rotation.

### 2.3. Additive Manufacturing of the Beam Specimens

The beam specimens were additively manufactured in an EOS M290 printer (EOS, Munich, Germany) using laser powder bed fusion (LPBF) technology. The cubic scaffold geometry includes a replicate of 105 × 17 × 13 cells to form the specimen’s length, width, and thickness, respectively. The average length is 151.9 mm, the average width is 25.25 mm, and the average thickness is 19.5 mm. The diagonal scaffold geometry includes a replicate of 57 × 9 × 7 cells to form the specimen’s length, width, and thickness, respectively. The average length is 149.3 mm, the average width is 24.2 mm, and the average thickness is 19.2 mm. The structures were oriented at an angle of 30 degrees on the 25 cm × 25 cm × 10 cm build platform. The support structure was created in Magics software to dissipate the heat from the newly printed layers such that thermally induced deformation during printing would be minimized. In addition, the support structure was optimized to reduce its amount and build time.

**Figure 2:**
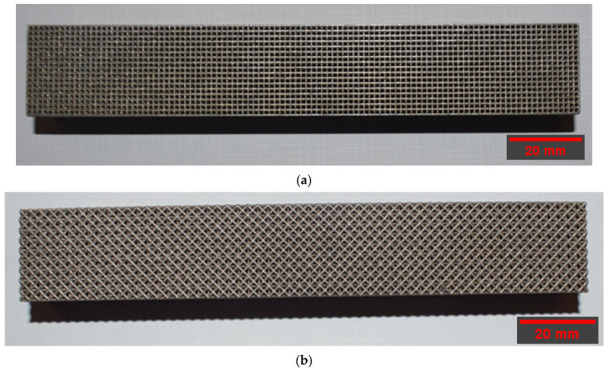
Beam specimens: (**a**) cubic, (**b**) diagonal.

A 400 W Ytterbium fiber laser with a wavelength of 1060 nm and beam diameter of 100 µm was used for the LPBF processing. Argon gas was used as an inert gas to keep the oxygen as low as 0.1% during printing to prevent oxidation. Gas atomized 316L SS powdered material with a particle size ranging from 15 to 40 microns was used. The default process parameters used were laser power of 195 W, laser scanning speed of 1083 mm/s, layer thickness of 20 µm, and hatch distance of 0.09 mm. The support structure was sintered in 40 µm layers with a laser power of 100 W and laser speed of 675 mm/s. Electrical discharge machining (EDM) was used to remove the scaffold from the substrate. Figure 2 depicts the 3D-printed cubic and diagonal beam specimens using LPBF after support material and build plate removal.

### 2.4. Flexural Testing of the BTE Scaffolds

Flexural experimental testing was conducted to validate the FEA results. The bending and shear moduli of the cubic and diagonal scaffolds were experimentally evaluated by conducting a three-point bending test. The tests have been conducted as per ASTM C 1674 and D 7264/D 7264 standards. The test setup includes a scaffold with rectangular geometry that rests on two supports and is loaded by means of a loading roller midway between the outer supports. The loading and support rollers have cylindrical contact surfaces of a radius of 15 mm to minimize stress concentration and prevent indentations at the loading and support locations.

The test machine was properly calibrated, and the load was applied at a constant rate of crosshead motion. The crosshead rate was selected so that the strain rate upon the specimen shall be of the order of 1.0 × 10^−4^ s^−1^. The crosshead rate was calculated as per the equation below:

(3) $$s=\epsilon *\frac{L^{2}}{6*d}$$

where *ϵ* is the desired nominal strain rate = 1.0 × 10^−4^ s^−1^, *d* is the specimen thickness (mm), *s* is the crosshead rate (mm/s), and *L* is the support span (mm). Using a strain rate of 1.0 × 10^−4^ s^−1^, span length of 120 mm, and height of 20 mm, the crosshead rate was calculated to be 0.72 mm/min. A 0.5 mm/min was chosen for better resolution as recommended by the ASTM standards, and relevant researchers [24].

The specimen deflection at the center of the loading span was measured using an internal linear variable differential transformer (LVDT) (Instron, Norwood, MA, USA) inside the platens and an external LVDT mounted on the loading plates. The deformation readings from both sources were compared, and no differences were found. Two specimens of each design were loaded to failure, and one was loaded in the linear elastic region. The number of specimens was sufficient as valid and consistent results were acquired. The load-deflection plot was generated from the experimental data for both scaffold designs.

The maximum stress for any point in the linear elastic region was calculated using the equation shown below:

(4) $$\sigma =\frac{3*P*L}{2*b*d^{2}}$$

where *σ* is the stress at the outer surface at mid-span (MPa), *P* is the applied force (N), *L* is the support span (mm), *b* is the width of the beam (mm), and *d* is the thickness of the beam (mm).

The maximum strain at the outer surface also occurred at mid-span and was calculated using the equation below:

(5) $$\epsilon =\frac{6*\delta *d}{L^{2}}$$

where *ϵ* is the maximum strain at the outer surface (mm/mm), *δ* is the mid-span deflection, *L* is the support span, and *d* is the specimen thickness.

**Figure 3:**
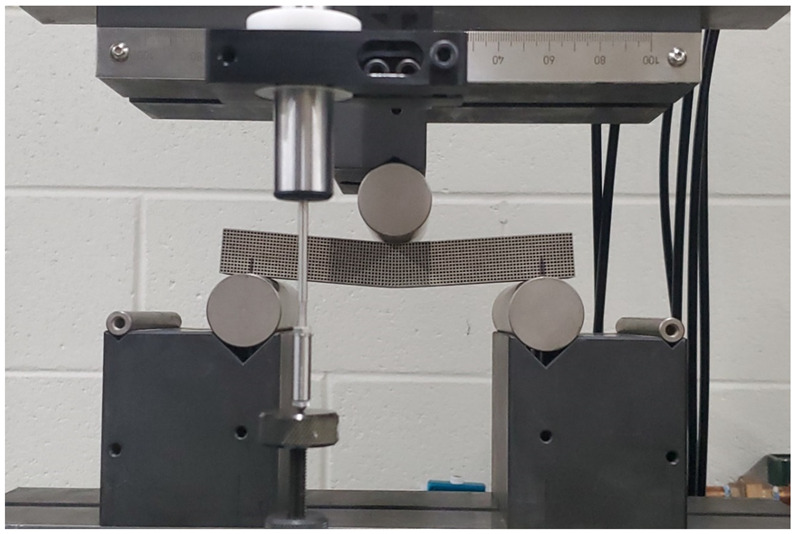
Flexure test for cubic scaffold.

The flexural (bending) modulus is calculated as the slope of the linear elastic line as per the equation below:

(6) $$E=\frac{\Delta \sigma }{\Delta \epsilon }$$

where Δ*σ* is the difference in flexural stress between two points in the linear elastic region, and Δ*ϵ* is the difference between two selected strain points in the linear elastic region. The experimental setup for the beam specimens is shown in Figure 3, respectively. To evaluate the shear modulus of the scaffolds using a three-point bending test, a strain rosette was mounted on the specimen and loaded elastically. The shear strain, as well as normal strains in the x and y directions, can be calculated using the three equations below:

(7) $$\epsilon_{a}=\epsilon_{x}\cos^{2}\theta_{1}+\epsilon_{y}\sin^{2}\theta_{1}+\gamma_{xy}\cos\theta_{1}\sin\theta_{1}\;$$

(8) $$\epsilon_{b}=\epsilon_{x}\cos^{2}\theta_{2}+\epsilon_{y}\sin^{2}\theta_{2}+\gamma_{xy}\cos\theta_{2}\sin\theta_{2}\;$$

(9) $$\epsilon_{c}=\epsilon_{x}\cos^{2}\theta_{3}+\epsilon_{y}\sin^{2}\theta_{3}+\gamma_{xy}\cos\theta_{3}\sin\theta_{3}\;$$

These equations can be solved simultaneously for *ϵ_x_*, *ϵ_y_*, and *γ_xy,_* knowing the values for the strains *ϵ_a,_* *ϵ_b,_* and *ϵ_c._* The arrangement of strain gauges used to measure those strains is referred to as 45°. Substituting *θ_1_* = −45°, *θ_2_* = 0°, and *θ_3_* = +45° in Equations (7)–(9), and solving for $\gamma_{xy}$ gives the following relationship:

(10) $$\gamma_{xy}=\epsilon_{a}-\epsilon_{c}$$

The first step in installing the strain gauge is preparing the surface of the specimen for strain gauge bonding. Five basic operations are included in the surface preparation. These are, in the usual order of execution: solvent degreasing, abrading, application of gauge layout lines, conditioning, and finally, neutralizing.

Initially, the gaging area was degreased with a solvent such as GC-6 Isopropyl Alcohol; then, preliminary dry abrading was conducted with 220 grit silicon-carbide paper to remove any surface oxidation. Final abrading was performed using 320-grit silicon-carbide paper on surfaces wetted with M-Prep conditioner A. Then, the gaging area was marked with layout lines to accurately locate and orient the staring gauge. After marking the layout lines, Conditioner A was applied repeatedly, and all the residue and conditioner were removed by slowly wiping the surface with a gauze sponge. In the final step, Neutralizer 5A was applied to bring the conditioned surface back to optimum alkalinity of 7.0 to 7.5 pH, which is considered appropriate for all strain gauges.

After preparing the surface, the gauge was installed and bonded to the specimen using M-Bond 200 adhesive. Next, M-Bond 200 catalyst was applied to expedite the hardening of the adhesive. The final step included the soldering process attaching the electrical wires to the installed strain gauge.

**Figure 4:**
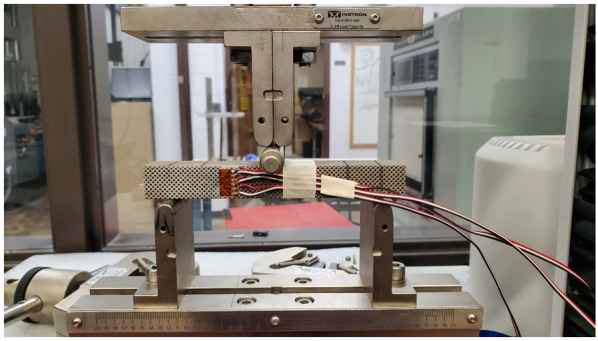
Bending test for shear modulus validation.

The wires were connected to a calibrated strain reader device, which gives strain values while loading the specimen. The specimens were loaded in three-point bending, as shown in Figure 4. The load was applied manually at small increments in the elastic zone. Three strain values were obtained at each load increment. After calculating the value of the shear strain “$\gamma_{xy}$” using Equation (10), the shear modulus “*G*” of the scaffold was obtained using the following equation:

(11) $$\tau =G\;\gamma_{xy}\;\;$$

where τ is the shear stress at the location where the gauge was mounted, *G* is the shear modulus, and $\gamma_{xy}\;$ is the shear strain.

The shear stress can be calculated using the following equation:

(12) $$\tau =\frac{VQ}{It}$$

where *V* is the shear force at the strain gauge’s location, *Q* is the first moment of area, *I* is the moment of inertia, and *t* is the beam thickness. Since the gauge was mounted at the center of the beam cross-section, the maximum value for *Q* was calculated and used in Equation (12).

## 3. Results and Discussion

### 3.1. FEA Results and Morphological Characterization

The data in Table 3 present the elastic properties for the diagonal and cubic designs from the numerical analysis. The same results were obtained and presented by [17] with a detailed interpretation of the findings.

The dry weighing and Archimedes techniques were used to determine the structure relative density of the manufactured scaffolds. Dry weighing of the samples was accomplished in normal room temperature and atmospheric conditions. The relative density of the cellular scaffolds was calculated by dividing the measured weight by the theoretical weight of the solid specimen. The theoretical weight was calculated using the theoretical density of the solid SS316L as suggested by the manufacturer, which equals 7.9 g/cm^3^. In the Archimedes technique, the scaffolds were weighed in dry and submerged conditions to the actual volume of the specimens. Comparisons between the manufactured scaffold structure’s relative density for each unit cell with the optimized scaffold designs are presented in Table 4. The average relative densities for cubic and diagonal scaffolds determined using dry weighing are 32.2% and 34.5%, respectively. The average relative densities for cubic and diagonal scaffolds determined using the Archimedes technique are 33.3% and 35.4%, respectively. The standard deviation is approximately 0.3% for the cubic scaffold and 0.2% for the diagonal scaffolds for both techniques. Comparing the manufactured scaffolds with optimized designs, the percentage error for the cubic and diagonal designs are approximately 5.0% and 6.6%, respectively.

### 3.2. Validating FEA Results by Experimental Testing

**Figure 5:**
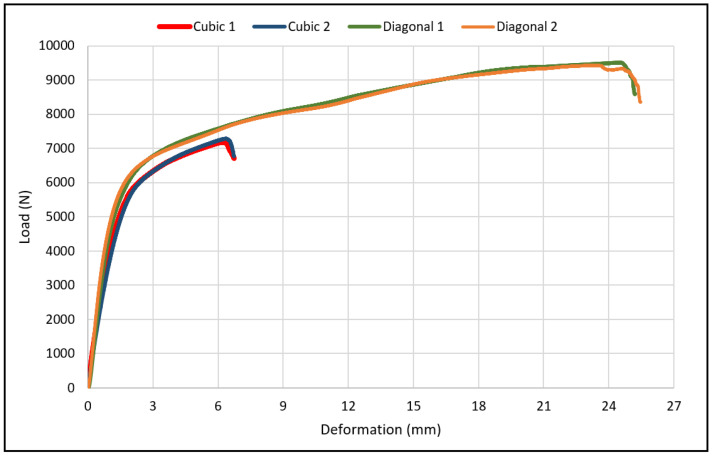
Flexural load-deflection curve for the cubic and scaffolds.

**Figure 6:**
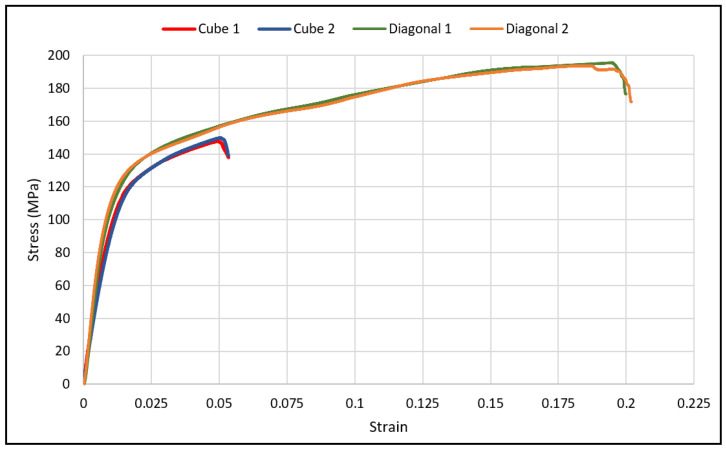
Flexural stress-strain curves for cubic and diagonal scaffolds.

**Figure 7:**
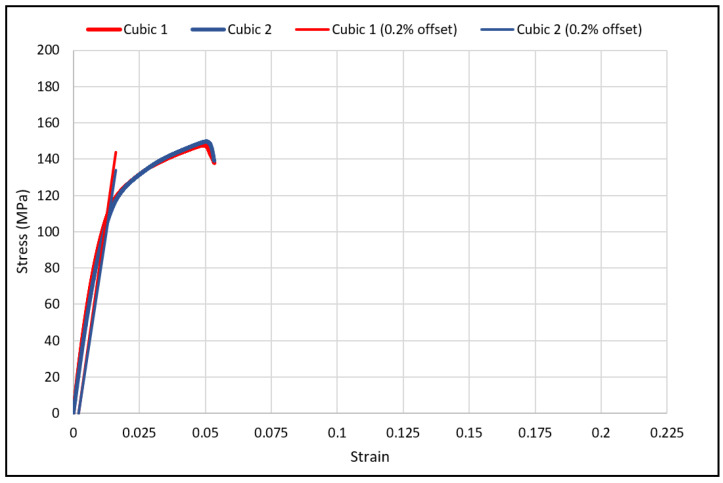
The 0.2% offset yield strength for cubic scaffolds.

**Figure 8:**
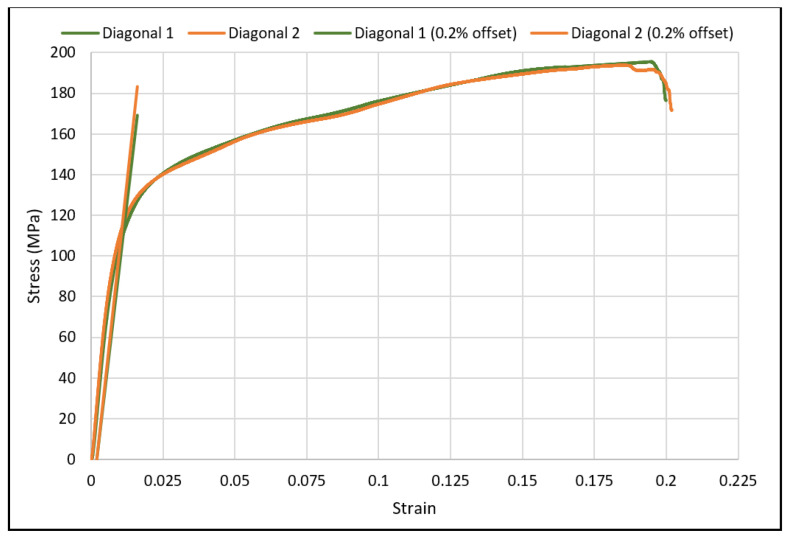
The 0.2% offset yield strength for diagonal scaffolds.

The load-deflection and the corresponding stress-strain curves for the cubic and diagonal scaffolds are presented in Figure 5 and Figure 6, respectively. The flexural properties of the cubic and diagonal scaffolds are presented in Table 5. The beam theory used to calculate stresses and strains from the load-deformation data assumes linear elastic behavior throughout the test. However, this assumption does not hold true beyond the point of yield; consequently, bending test results tend to overestimate ultimate material strength. The yield strength (*σ_fy_*) and yield strain (*ϵ_y_*) were determined using the 0.2% strain offset method, as shown in Figure 7 and Figure 8. Due to the overestimation of the stress in the post-yield region, the energy to failure results rather than energy to fracture are presented in this study, which represent estimates rather than accurate values. The slope of the linear elastic line of the stress-strain curve represents the bending modulus of the scaffold. The average experimental bending moduli for the cubic and diagonal scaffolds were 9.49 GPa and 14.76 GPa, respectively. The percentage errors in the bending modulus between the numerical and experimental results for the cubic and diagonal scaffolds were 7.77% and 7.92%, respectively. Such variation can be explained by the difference in relative density between the manufactured specimens and CAD design. Hence, it is concluded that the experimental values for the bending moduli of the tested scaffolds are in agreement with the numerical results. For the diagonal scaffold, the 45 degrees orientation of the struts has a significant impact on all determined flexural properties.

**Figure 9:**
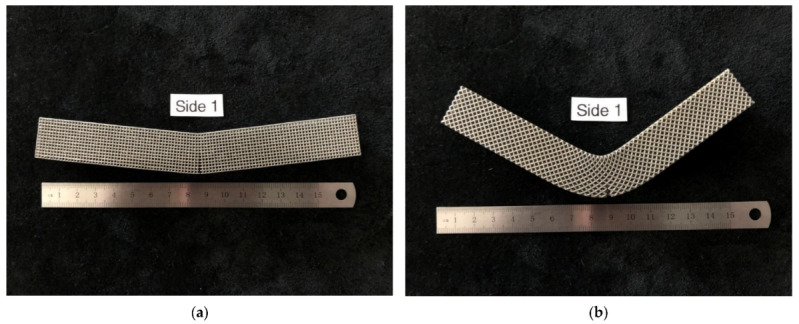
Deformed specimens (**a**) cubic scaffold, (**b**) diagonal scaffold.

The bending modulus was 36% higher in the diagonal scaffold than in the cubic scaffold. Similarly, the ultimate flexural strength was 24% higher for the diagonal scaffold than for the cubic scaffold. When comparing the yield strength and yield strain of both designs, no significant differences were observed. The yield strength of the diagonal scaffold was slightly higher than the cubic scaffold, whereas the yield strain was a bit lower for the diagonal scaffold than the cubic scaffold. Substantial differences were observed when comparing the strain at ultimate and energy absorption. The ultimate strain for the diagonal scaffold was 280% higher than the cubic scaffold, and the energy absorption was 430% higher in the diagonal unit cell design compared with the cubic design. Hence, the diagonal scaffold was observed to be significantly tough and can absorb substantial energy before fracture in bending. The deformed shapes for both scaffolds in bending are shown in Figure 9.

The stress-strain curve for the diagonal scaffold resembles the behavior of a cortical bone loaded longitudinally. In contrast, the stress-strain curve for the cubic scaffolds is comparable to a cortical bone loaded transversely. The cortical bone is classified as a relatively ductile material for longitudinal loading since its ultimate strain for longitudinal loading is substantially larger than its yield strain. However, it is relatively brittle for transverse loading. The bending test results for the cubic and diagonal scaffolds fairly agree with the cortical bone behavior for both directions of loading.

**Figure 10:**
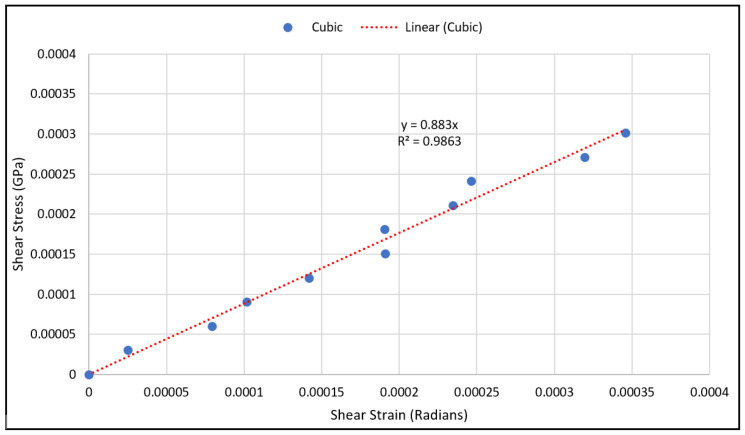
Shear stress vs. shear strain data points for the cubic scaffold.

**Figure 11:**
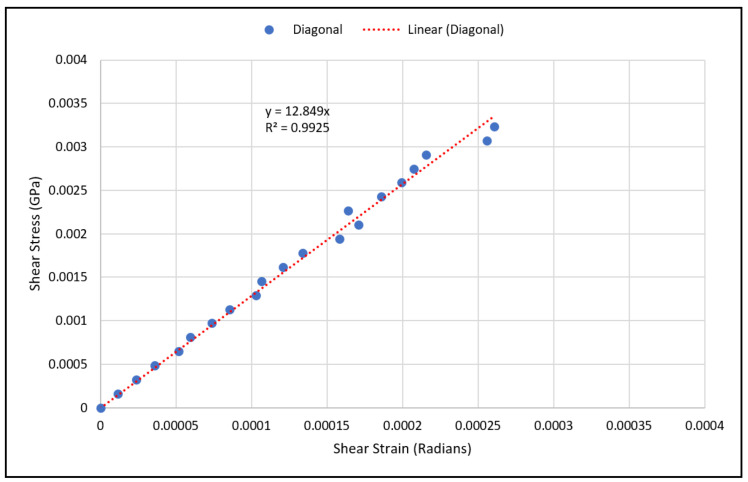
Shear stress vs. shear strain data points for the diagonal scaffold.

As for validating shear modulus results, the shear strain has been calculated using Equation (10) at various load values, and the shear stress has been calculated using Equation (12) from the measured strain gauge readings. The shear stresses with the corresponding strains were plotted using a scatter plot, and a linear regression was used to generate the linear equation that best fit the plotted data, as shown in Figure 10 and Figure 11. The linear relation represents an estimate of the linear elastic portion of the shear stress-shear strain curve for both scaffold designs in which the slope is the shear modulus. The experimental values for the shear moduli for the cubic and diagonal scaffolds were 0.883 GPa and 12.85 GPa, respectively. Since the scaffolds have a course microstructure for the size of strain gauge used, strain in the scaffolds may not be completely transmitted to the grid of the gauge, thus, impacting test results. It is important to note here that the strain gauge readings were consistent for the diagonal scaffold throughout the test, even at higher loads. However, the strain readings for the cubic scaffolds were consistent at very small loads and varied significantly at higher loads. The reason could be that the strain gauge surface is fully in contact with the angled struts in the diagonal scaffold, while in the cubic scaffold, the gauge pores and struts are in contact with the strain gauge. Another reason is that the strain gauge is more reliable in measuring shear strains in the diagonal unit cell as opposed to the cubic cell due to the fact that the maximum shear stress occurs at 45 degrees in which the diagonal struts are oriented.

### 3.3. Characterization of Strut Failure Due to Bending

**Figure 12:**
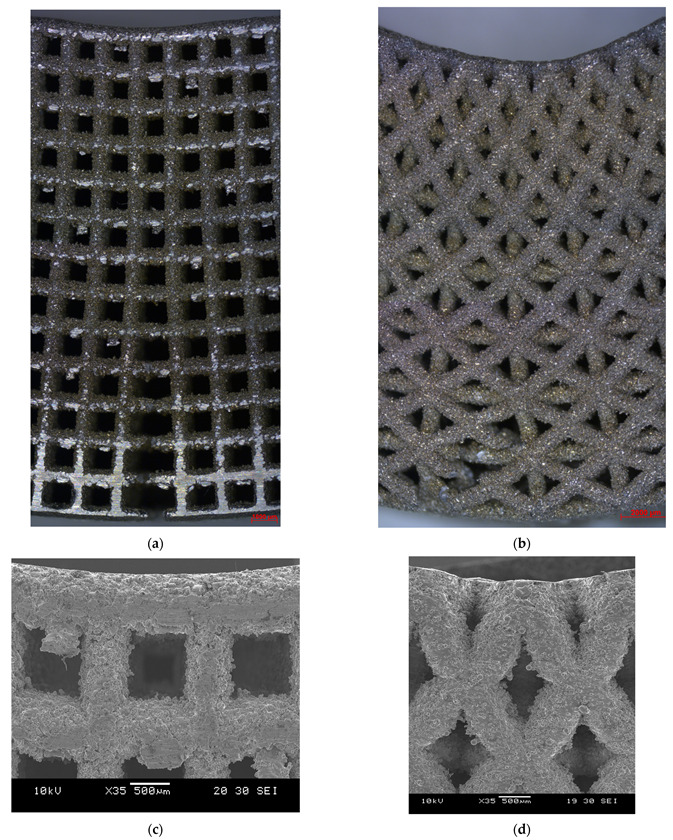
Bent specimens: (**a**) cubic (optical microscope), (**b**) diagonal (optical microscope), (**c**) cubic (SEM), and (**d**) diagonal (SEM).

As expected, the fracture of the specimens occurred in the middle of the specimen, where maximum bending stress exists. The bent specimens are shown in Figure 12. The pores at the top side of the diagonal scaffold were heavily compressed in comparison with the cubic scaffold. This is due to the extensive plastic deformation occurring in diagonal scaffolds and the rapid fracture of struts on the tension side of the cubic scaffold.

**Figure 13:**
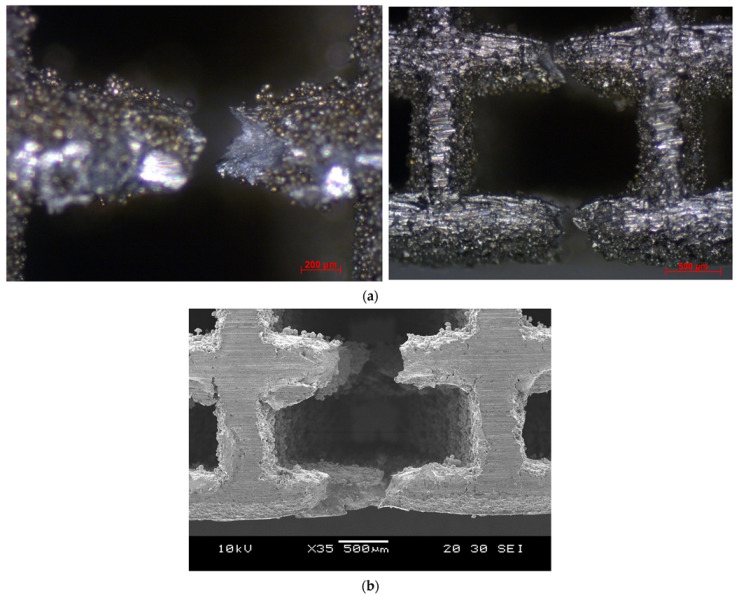
Necking in strut at the tension side (**a**) cubic (optical microscope), (**b**) cubic (SEM).

**Figure 14:**
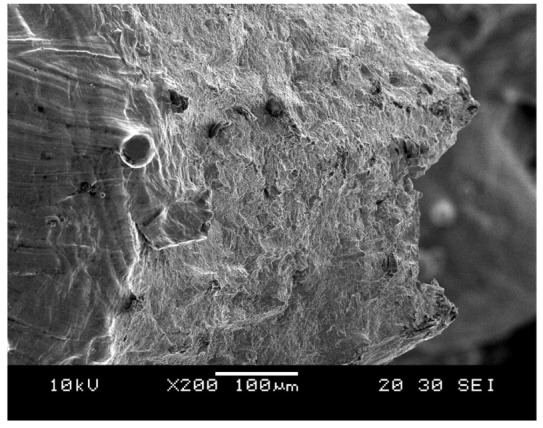
Rough and dull fractures surface for cubic scaffold (SEM).

The failure in struts in tension showed signs of ductility as necking was observed in fractured struts, as shown in Figure 13. In addition, the fracture surface was observed to be rough and dull as opposed to being smooth and bright like in brittle fractures, as shown in Figure 14.

**Figure 15:**
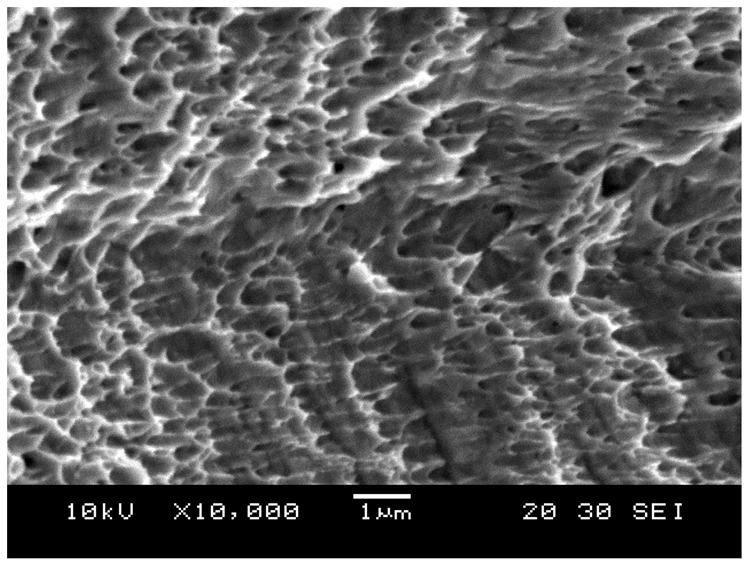
Dimple fracture.

Moreover, the image presented in Figure 15 was taken using SEM at ×10,000 magnification, which shows depressions in the microstructure. These depressions are called dimples. These dimples occur from microvoid emergence in places of high localized plastic deformation, which is also a sign of ductility in the material. The dimples were observed to be elongated instead of a complete rim due to the effect of shearing stress.

### 3.4. Comparison of the Mechanical Properties between Bone Scaffolds and Cortical Bone

Comparisons between the elastic stiffnesses of the cubic and diagonal scaffold with the cortical bone properties are presented in Table 6. The compressive modulus of both scaffolds falls in the elastic modulus range elastic of cortical bone loaded longitudinally. The bending modulus of the diagonal scaffold falls in the cortical bone range loaded longitudinally, while the bending modulus of the cubic scaffold is slightly lower than the cortical bone modulus. The diagonal scaffold shear modulus is substantially higher than the shear modulus of the cubic scaffold as well as the cortical bone.

In addition, the values for compressive yield strength, flexural yield strength, ultimate flexural strength, and energy absorption of both scaffold designs were compared with the cortical bone properties as presented in Table 7. The compressive yield and ultimate strength values of the cubic scaffold fall in the range of cortical bone loaded longitudinally. In comparison, the diagonal scaffold exhibited less compressive yield strength than the cortical bone loaded longitudinally. However, the diagonal scaffold compressive yield strength was observed to be comparable to the cortical bone loaded transversely. The flexural yield strength of both scaffold designs falls in the range of flexural yield strength values for cortical bone loaded longitudinally. As previously stated, the energy absorbed by the cubic scaffold was observed to be comparable to the cortical bone loaded transversely, while the energy absorbed by the diagonal scaffold was observed to be comparable to the cortical bone loaded longitudinally.

## 4. Conclusions

This research study presented the experimental validation and evaluation of the bending properties of optimized BTE scaffolds with an elastic modulus that is equivalent to the young’s modulus of the cortical bone. The morphological properties of the additively manufactured specimens were evaluated. The bending modulus of the cubic and diagonal scaffolds were experimentally investigated and found to agree with the numerical results. The diagonal scaffold’s ultimate strain was significantly higher than the cubic scaffold; hence, the diagonal scaffold was substantially tougher with considerably higher energy absorption before fracture. The shear modulus of the diagonal scaffold was significantly higher than the cubic scaffold. Finally, a comparison of the mechanical properties of the studied BTE scaffolds with the cortical bone properties under longitudinal and transverse loading was presented. Future work includes investigating the fatigue strength and comparing the fatigue behavior between the cubic and diagonal scaffold designs under cyclic loading. This research study can be further improved by conducting in vivo/in vitro studies on the designed scaffolds to investigate bone tissue regeneration aspects, including biocompatibility, surface bioactivity, cells adhesion and proliferation, osteogenic differentiation, osteoblasts culture, and scaffolds degradation.

## Figures and Tables

**Table 1:** Mechanical properties of 3D-printed SS316L [23].

Mechanical Property	Values Used in FEA Model
Elastic Modulus (E) GPA	190 ± 45
Ultimate Tensile Strength (UTS) MPa	671 ± 33
Yield Strength (YS) MPa	560 ± 25
Elongation %	24 ± 0.8

**Table 2:** Geometrical parameters of the optimized BTE scaffolds.

Unit Cell Type	Pore Size (Micron)	Cell Size (Micron)	Strut Size (Micron)
**Cubic**	798	1444	646
**Diagonal**	812	2616	734

**Table 3:** Elastic properties from the FEA.

Cell Type	Young’s Modulus (GPa)	Bending Modulus (GPa)	Shear Modulus (GPa)
**Cubic**	15	10.29	1.39
**Diagonal**	15	16.03	14.52

**Table 4:** Comparison in structure relative densities between the CAD file and manufactured scaffolds.

Unit Cell Design	Optimized Design (%)	Manufactured Scaffold (%)	
		Dry Weighing	Archimedes
**Cubic**	34.50	32.00	33.22
		32.57	33.67
		32.14	33.09
**Diagonal**	37.39	34.72	35.61
		34.29	35.15
		34.39	35.45

**Table 5:** Flexural properties of the cubic and diagonal scaffolds.

Cell Type	Bending Modulus (*E_b_*) (GPa)	Flexural Yield Strength (*σ_fy_*) (MPa)	Strain at Yield (*ϵ_y_*) (%)	Flexural Ultimate Strength (*σ_fu_*) (MPa)	Strain at Ultimate (*ϵ_u_*)	Energy to Ultimate Strain (MJ/m^3^)
Cubic 1	9.52	107.1	1.27	147.6	4.93	6.34
Cubic 2	9.45	106.4	1.32	149.7	5.05	6.30
Diagonal 1	14.69	111.6	1.13	195.6	19.51	33.4
Diagonal 2	14.83	114.4	1.08	193.7	18.43	33.70

**Table 6:** Comparison between the elastic stiffnesses of the cubic and diagonal scaffold with the cortical bone properties.

	Cubic Scaffold		Diagonal Scaffold		Cortical Bone	
	FEA	Exp.	FEA	Exp.	Longitudinal	Transverse
**Compressive Modulus (GPa)**	15.0 ^a^	14.76 ^a^	15.0 ^a^	14.29 ^a^	15.0 ^b^	11.5 ^c^
**Bending Modulus (GPa)**	10.29 ^a^	9.49	16.03 ^a^	14.76	12.8–19.3 ^d^	4.7–7.9 ^d^
**Shear Modulus (GPa)**	1.39 ^a^	0.883	14.52 ^a^	12.85	3.3–6.1 ^e^	N/A

^a^ [17]; ^b^ [25]; ^c^ [26]; ^d^ [27]; ^e^ [28].

**Table 7:** Comparison of mechanical strength between bone scaffolds and cortical bone.

	Cubic Scaffold	Diagonal Scaffold	Cortical Bone	
			Longitudinal	Transverse
**Compressive Yield Strength (MPa)**	122 ^a^	75 ^a^	99–130 ^b^	21–64 ^c^
**Compressive Strength (MPa)**	138 ^a^	133 ^a^	131–174 ^b^	52–78 ^c^
**Flexural Yield Strength (MPa)**	107	113	105–117 ^d^	43–63 ^d^
**Ultimate Flexural Strength (MPa)**	148	195	120–150 ^d^	43–71 ^e^
**Energy to Ultimate (MJ/m^3^)**	4.97	33.55	10.5–24.8 ^e^	0.25–1.53 ^e^

^a^ [17]; ^b^ [28]; ^c^ [29]; ^d^ [30]; ^e^ [27].
